# To Each Their Own: The Impact of Regulatory Focus on Consumers’ Response to Online Information Load

**DOI:** 10.3389/fnins.2022.757316

**Published:** 2022-04-18

**Authors:** Minjing Peng, Zhicheng Xu, Haiyang Huang

**Affiliations:** School of Economics and Management, Wuyi University, Jiangmen, China

**Keywords:** information load, regulatory focus, event-related potentials, P2, N2, P3

## Abstract

Contrary to the common view that more information is always better, too much information can damage decision quality. Building on existing literature, this study identified regulatory focus as a critical factor influencing the effect of information load (IL) on online consumer decisions and used event-related potentials (ERPs) to uncover its underlying neural mechanism. Behavioral data showed that promotion-focused participants would spend less time making purchasing decisions in the low IL condition than in the high IL condition. However, no significant difference was found for prevention-focused participants. In contrast to the high IL condition, ERP data indicated that the low IL condition recruited more attentional resources at the early stage of rapid automated processing (larger P2 component), leading to reduced long-term memory conflict (smaller N2 component), and resulting in enhanced decision confidence (larger P3 component) for those with a promotion focus. However, we observed either weakened or even opposite outcomes for those with a prevention focus. These findings generally shed light on when e-retailers should provide large/small amounts of product information in online environments.

## Introduction

Information provision has always been a challenge in marketing practice and research. Traditional marketing believes that rich information may play a vital role in high-quality purchase decisions (Alba et al., 1997). The greatest strength of electronic retailers is the capacity to provide consumers with massive information at little cost, which can reduce the effort of searching for information, reduce information asymmetry and make informed buying decisions (Chen et al., 2009). According to the theory of information overload, however, when input surpasses the processing capacity, it will damage decision quality, resulting in decision-making delay, reduces decision satisfaction, and causes more regret (Jacoby et al., 1974; Lee and Lee, 2004; Inbar et al., 2011; Chernev et al., 2015; Roetzel, 2018).

Considering the controversial views regarding the impact of information load (IL) on consumer decisions, the present study attempts to disclose the contingency conditions on the preferences for consumers’ IL by ascertaining a consumer-level factor (i.e., regulatory focus).

The regulatory focus theory indicates that individuals will show diverse ways of achieving their objectives (Higgins, 1997). Previous studies have distinguished between two different motivational orientations: promotion-focused and prevention-focused (Crowe and Higgins, 1997). More specifically, promotion-focused individuals concentrate on pursuing opportunities, favorable outcomes, and the goals of the maximization of achievement (Rhee and Fiss, 2014; Zhang et al., 2014). In contrast, prevention-focused individuals care more about security, negative results, and the aim of the minimization of loss (Wan et al., 2009; Higgins and Cornwell, 2016).

The regulatory focus has been shown to influence consumer decision-making. During the information search phase, promotion-focused consumers will generate more diversified consideration sets than prevention-focused consumers (Wang and Lee, 2006; Pham and Chang, 2010). In the information processing phase, promotion-focused participants prefer an exploratory way, while prevention-focused participants favor a cautious approach (Förster et al., 2003). Therefore, promotion-focused individuals rely on heuristic strategies to simplify the evaluation process when confronted with product information. Prevention-focused individuals, by contrast, tend to adopt a systematic approach to enhance the accuracy of their decisions (Wan et al., 2009). The impact of regulatory focus upon consumers’ online decisions for products under different IL conditions, however, has not been examined. The present study attempts to address this gap by drawing upon the regulatory fit view.

The regulatory fit view, concerning fit with the individuals’ self-regulatory orientation and the task at hand, suggests that such a fit can enhance persuasion and affect consumer decisions (Higgins, 2000). That is because such a fit will result in consumers experiencing processing fluency (Lee and Aaker, 2004; Lee and Labroo, 2004; Cesario and Higgins, 2008; Alter and Oppenheimer, 2009). More importantly, such positive impacts of regulatory fit have been documented for engagement strength, decision confidence, willingness to buy, and consumer preference (Avnet and Tory Higgins, 2003; Higgins et al., 2003; Wan et al., 2009; Li et al., 2020; Liu et al., 2020). Thus, we speculate that IL conditions and individuals’ regulatory focus may have a regulatory fit in the decision process. Specifically, prevention-focused motivation is related to cautious and risk-averse behavior, which may evoke a preference for specific and detailed information. Prevention-focused individuals may, therefore, prefer the high IL condition over the low IL condition. In contrast, promotion-focused individuals prefer speed over accuracy (Förster et al., 2003). Because less information reflects rapid heuristic processing, promotion-focused individuals might prefer the low IL condition over the high IL condition. In turn, the presence of such a fit will trigger a more favorable decision experience than would occur in the absence of such a fit. However, there is no direct behavioral or psychological evidence to verify this hypothesis.

The present study adopted event-related potentials (ERPs) to explore how those with a promotion or prevention focus respond variously to IL. Based on previous research regarding buying decisions, this study focused on the P2, N2, and P3 components.

The P2 is a positive-going component over the prefrontal-central cortex (Polezzi et al., 2008). Previous studies have consistently found that a larger P2 amplitude would be found when more attentional resources were devoted (Huang and Luo, 2006; Mercado et al., 2006; Yuan et al., 2007). For example, negative framing messages automatically mobilized more attentional resources and induced a larger P2 amplitude than positive framing messages (Jin et al., 2017). Furthermore, situations with reviewer photos would recruit more attentional resources and induce a larger P2 amplitude than situations without reviewer photos (Tang and Song, 2021). According to regulatory fit, consumers would devote more attentional resources to the cognitive processing of product information when the task at hand matched with individuals’ regulatory focus (Higgins, 2000; Lee and Aaker, 2004; Cesario and Higgins, 2008). Thus, we speculate that more attentional resources will be invested if IL matches with consumers’ regulatory focus, as reflected by the larger P2. Specifically, the P2 amplitudes induced by the low IL condition are more positive than by the high IL condition for promotion-focused consumers, whereas the reverse holds for prevention-focused consumers.

The N2 is a negative-going component with a frontal-central cortex distribution peaking at around 250–350 ms (Folstein et al., 2008). Prior studies indicated that it was relevant to conflict and mismatch (Van Veen and Carter, 2002). For example, more negative N2 amplitudes emerged when the second stimuli did not match the physical characteristics of the first stimuli concerning color or position based on the S1–S2 paradigm (Wang et al., 2004; Mao and Wang, 2008). Besides the conflicts between these physical properties, the N2 could also be elicited by perception conflicts (Ma et al., 2007). For example, a higher cognitive conflict would be observed in the counter-conformity decisions, and then a larger N2 amplitude would be evoked (Gajewski et al., 2016). Conversely, Shang et al. (2017) suggested no conflict would be produced when consumers perceived a more excellent brand extension fit, which can be revealed in a smaller N2 amplitude. In addition, Achtziger et al. (2014) showed that participants who over-valued new information in the belief-updating economic decisions were less sensitive to conflict detection, as reflected by the N2. According to regulatory fit, consumers would produce a sense of fluency and perceive a smaller decision conflict when the task at hand matches with individuals’ regulatory focus (Sellier and Chattopadhyay, 2009). Thus, we assume that more cognitive conflicts will be caused and elicit a larger N2 amplitude in the decision process if IL mismatches with consumers’ regulatory focus. More specifically, the high IL condition will induce a larger N2 amplitude compared to the low IL condition for promotion-focused consumers. In contrast, the opposite results will be found for prevention-focused consumers.

The P3 is an ERP component maximal over the central-parietal cortex and typically peaks at approximately 300–500 ms after stimulus presentation (Folstein et al., 2008). Prior studies suggested that this component exhibited a high sensibility to information about rewards and punishments. For example, Yeung and Sanfey (2004) showed that the P3 was sensitive to the size of the reward in a gambling game. Furthermore, past research has shown a positive correlation between decision confidence and the P3 amplitude (Nieuwenhuis et al., 2005). For example, in consistent situations, the participants would perceive less decision difficulty and be more confident, as reflected by a larger P3 amplitude (Xie et al., 2016). According to regulatory fit, consumers would increase confidence and perceive less difficulty when the task at hand matches with individuals’ regulatory focus (Higgins et al., 2003). Thus, we speculate that the decision difficulty will decrease, and the confidence will increase if IL matches with consumers’ regulatory focus, as evidenced by a larger P3 amplitude. Concretely, the P3 amplitudes elicited by the low IL condition will be more positive than by the high IL condition for those with a promotion focus, while the reverse holds for those with a prevention focus.

Based on the above discussions, we propose the following assumptions: the increased P2 and P3, and an attenuated N2 will be elicited in the low IL condition than in the high IL condition for promotion-focus consumers, whereas the reverse holds for prevention-focused consumers.

## Materials and Methods

### Participants

A total of 40 right-handed students (50% male; mean age = 23.5) with normal vision were recruited from Wuyi University through web advertisements and were paid $5 each for taking part in the study. All of them reported no history of mental or neurological disorders. The participants were called upon to give written informed consent at the start of the experiment under the Declaration of Helsinki.

### Materials and Pretest

Unlike previous IL studies, which chose durable goods as experimental materials (Lee and Lee, 2004; Chen et al., 2009; Sicilia and Ruiz, 2010), we chose fruits (non-durable products) as the material for our study. Previous studies have shown that consumers may be very interested in receiving detailed product information for durable goods (Burke, 2002). However, recent studies have indicated that when purchasing fruits, consumers may also express an interest in fruit origin information (Fernández-Serrano et al., 2020). Thus, we included the climate, temperature, precipitation, and sunshine suitability of the origin besides the brand and price labels.

Based on previous studies (Sicilia and Ruiz, 2010), we developed two versions of the material to manipulate the IL condition. Specifically, the ultimate IL for each condition (six for the low IL condition and twelve for the high IL condition) was established through a pretest. In this pretest (*n* = 201), we used a 5-point Likert scale adapted from Lee and Lee (2004) to determine the level of perceived IL (i.e., “There were many characteristics of fruits to consider”). An independent-sample *t*-test indicated a significant difference [*M*_*low*_ = 2.90 vs. *M*_*high*_ = 3.85; t(199) = −15.50; *p* < 0.001] in perceiving IL levels between the two IL conditions, which suggested that there is more information needed to be addressed for participants in the high IL condition. All pictures were processed to maintain consistency in text style, lightness, and saturation.

For regulatory focus, participants were asked to complete the Regulatory Focus Questionnaire (Higgins et al., 2001), consisting of items of both promotion (e.g., “Do you often do well at different things that you try?” 1 = never or seldom, 5 = very often; α = 0.73) and prevention (e.g., “Do you often obey rules which were established by your parents?”; 1 = never or seldom, 5 = very often; α = 0.80) foci. The total score of the prevention subscale was subtracted from the total score of the promotion subscale to generate a difference score. A median split of the difference scores was applied to classify participants as primary promotion- or prevention-focused compared to others (Higgins et al., 2001). Finally, there were 19 participants in the prevention-focused group (47.37% female; Mean age = 23) and 19 participants in the promotion-focused group (52.63% females; Mean age = 24). Two participants, whose scores were equal to the median, were excluded.

### Procedure

**FIGURE 1:**
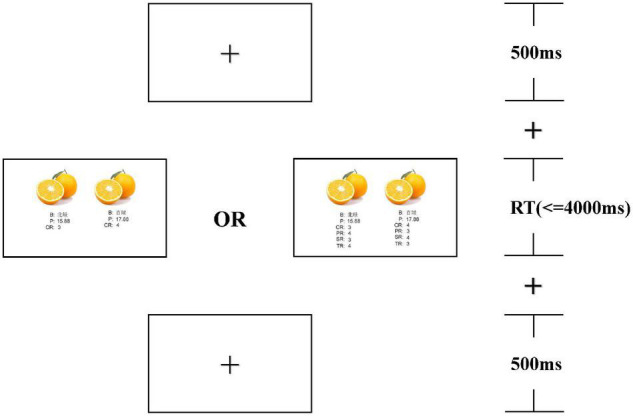
Experimental procedure (B for the brand, P for the price, CR for climate rating, PR for precipitation rating, SR for sunshine rating, and TR for temperature rating).

Participants were seated in a comfortable armchair in a soundproof lab. They were instructed to receive guidance on current tasks and imagine searching for fruits on the e-commerce platform upon entering the study. The experiment was comprised of two blocks, each containing 100 trials. As illustrated in Figure 1, at the beginning of each trial, a “+” was fixed in the center of the screen for 500 ms, then, they had a decision-making task to complete. During each decision task, they were required to choose between the left and the right fruits. Participants had up to 4,000ms to decide by pressing a button. The response-to-hand assignments were counterbalanced across individuals such that half of them were informed to press “A” for “Buy the fruit on your right” and “B” for “Buy the fruit on your left”; while the mode was reversed for the others. All decision tasks were presented randomly in the experiment. We used E-prime 3.0 software to present the program and record the data. The formal experiment started after four practice trials.

### Electroencephalogram Recording and Analysis

The electroencephalogram (EEG) was recorded with a Brain Product Amplifier (Brain Products GmbH, Munich, Germany), containing 32 Ag/AgCl electrodes with a sampling rate of 500 Hz. The amplifier band-pass was 0.01–100 Hz, and the linked mastoid served as an online reference. We ensured that the impedance of all electrodes was kept below 10 kΩ throughout the experiment.

We used EEGLAB to deal with the offline EEG data, with recordings re-referenced to the “infinity” reference provided by the reference electrode standardization technique (REST; Yao, 2001). Independent Component Analysis was applied to remove artifacts embedded in the data (EMG, eye blinks, and eye movements). The EEG data were segmented for the epoch from 200 ms before the onset of the stimulus and lasted 1,000 ms, with the 200 ms pre-stimuli interval as a baseline. In addition, a low-pass filter at 30 Hz was used to filter the EEG digitally, and trials exceeding ±75 μV would be removed.

The ERP results were developed for four experimental conditions separately (IL: high vs. low × regulatory focus: promotion vs. prevention). It should be noted that regulatory focus is a between-subjects variable and IL is a within-subjects variable. As we expected, P2, N2, and P3 components were successfully elicited in our results. Based on the visual observation of the grand-averaged ERPs waveforms and past research on purchase decisions (Ma et al., 2008; Gajewski et al., 2016; Jin et al., 2020; Tang and Song, 2021), five electrodes (F3, F4, FC1, FC2, and Fz) were selected for P2 and N2 analysis, and five additional electrodes (CP1, CP2, P3, P4, and Pz) were chosen for the P3 analysis. The P2 was analyzed as the peak amplitude in the time window of 130–200 ms after onset. The N2 was analyzed as the peak amplitude in the 200–300 ms time window. Meanwhile, the P3 was analyzed as the peak amplitude in the time window of 300–450 ms. The Greenhouse–Geisser correction was applied for violation of the sphericity assumption.

## Results

### Behavioral Data

A two-way 2 (IL: low vs. high) × 2 (regulatory focus: promotion vs. prevention) mixed repeated measure ANOVA was performed for the response times (RTs). We used SPSS 25.0 for statistical tests. The results demonstrated a significant main effect of IL [*F* (1, 18) = 28.791, *p* < 0.001, *η*^2^P = 0.113]: the RTs for the high IL condition (*M* = 1,682 ms, *SD* = 46) were longer than the low IL condition (*M* = 1,595 ms, *SD* = 44). Furthermore, the main effect of regulatory focus was significant [*F* (1,18) = 5.787, *p* < 0.05, *η*^2^P = 0.243]: the RTs for prevention-focused individuals (*M* = 1,786 ms, *SD* = 66) were longer than for promotion-focused individuals (*M* = 1,491 ms, *SD* = 84). Importantly, the interaction between IL and regulatory focus was also significant [*F* (1, 18) = 5.147, *p* < 0.05, *η*^2^P = 0.222]. A simple effect analysis showed that the RTs for the low IL condition (*M* = 1,420 ms, *SD* = 78) were significantly shorter than those for the high IL condition (*M* = 1,563 ms, *SD* = 91) for promotion-focused consumers [*F* (1, 18) = 28.332, *p* < 0.001, *η*^2^P = 0.612], while the contrast between the low IL condition and the high IL condition for prevention-focused consumers was not significant [*F* (1, 18) = 0.979, *p* > 0.05, *η*^2^P = 0.052].

### Event-Related Potential Data

**FIGURE 2:**
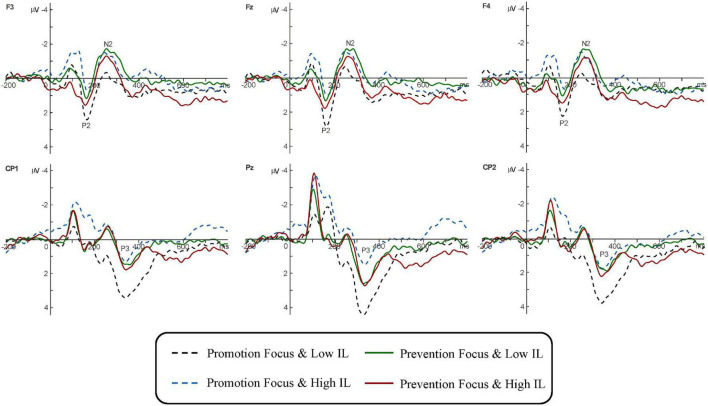
Grand-averaged ERP elicited by four conditions at representative electrodes F3, Fz, F4, CP1, Pz, and CP2.

Figure 2 illustrates the grand-averaged ERP evoked by four conditions. A 2 (IL) × 2 (regulatory focus) × 5 (electrode) mixed repeated measure ANOVA was conducted for the peak amplitudes of the P2, N2, and P3.

**FIGURE 3:**
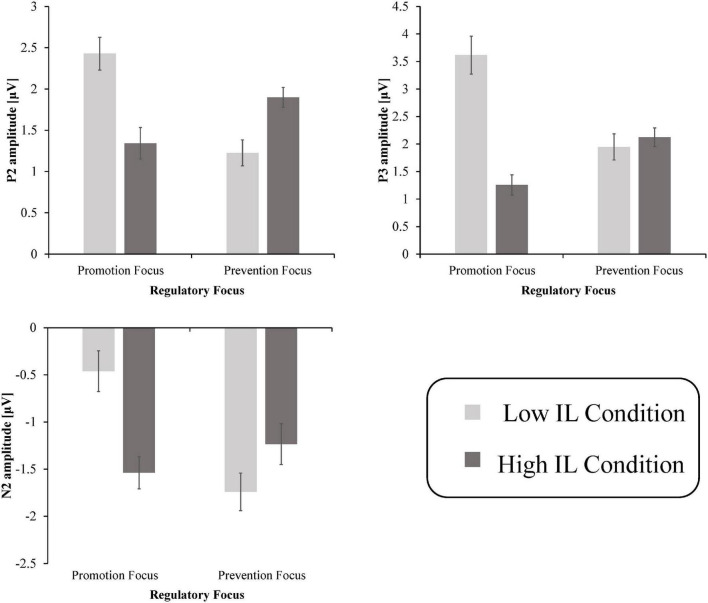
Mean peak amplitudes of the P2, N2 in F3/F4/Fz/FC1/FC2, and P3 in CP1/CP2/P3/P4/Pz. The error bars refer to SEM.

With regard to the P2, the results showed that there were no significant main effects of regulatory focus [$F\,\left(1,18\right)=4.07,p>0.05,\eta_{\text{P}}^{2}=0.184$], IL [$F\,\left(1,18\right)=1.783,p>0.05,\eta_{\text{P}}^{2}=0.09$], or electrode [$F\,\left(4,72\right)=2.791,p>0.05,\eta_{\text{P}}^{2}=0.134$]. The interaction between IL and regulatory focus was significant [$F\,\left(1,18\right)=78.45,p<0.001,\eta_{\text{P}}^{2}=0.813$], as shown in Figure 3. A simple effect analysis indicated that the P2 amplitudes in the low IL condition (*M* = 2.428μV,S.E. = 0.198) were significantly larger than in the high IL condition (*M* = 1.342μV,S.E. = 0.157) for promotion-focused consumers [$F\,\left(1,18\right)=55.568,p<0.001,\eta_{\text{P}}^{2}=0.755$], and the reverse held (M_low_ =  1.226μV,S.E_.low_ = 0.191;M_high_ = 1.899μV,S.E_.high_ = 0.121) for prevention-focused consumers [$F\,\left(1,18\right)=9.753,p<0.05,\eta_{\text{P}}^{2}=0.351$]. However, there were no interaction effects of regulatory focus × electrode [$\text{F}\,\left(4,72\right)=0.538,p>0.05,\eta_{\text{P}}^{2}=0.029$], IL × electrode [$\text{F}\,\left(4,72\right)=0645,p>0.05,\eta_{\text{P}}^{2}=0.062$], or regulatory focus × IL × electrode [$\text{F}\,\left(4,72\right)=1.259,p>0.05,\eta_{\text{P}}^{2}=0.065$].

As for the N2, the ANOVA demonstrated that there were no significant main effects of regulatory focus [$\text{F}\,\left(1,18\right)=3.5,p>0.05,\eta_{\text{P}}^{2}=0.163$], IL [$\text{F}\,\left(1,18\right)=3.697,p>0.05,\eta_{\text{P}}^{2}=0.17$], or electrode [$\text{F}\,\left(4,72\right)=2.246,p>0.05,\eta_{\text{P}}^{2}=0.111$]. Importantly, the interaction between IL and regulatory focus was significant [$\text{F}\,\left(1,18\right)=49.985,p<0.001,\eta_{\text{P}}^{2}=0.735$], as shown in Figure 3. A simple effect analysis indicated that the N2 amplitudes in the high IL condition (*M* = −1.539μV,S.E. = 0.2) were more negative than in the low IL condition (*M* = −0.461μV,S.E. = 0.216) for promotion-focused consumers [$F\,\left(1,18\right)=31.121,p<0.001,\eta_{\text{P}}^{2}=0.634$], and we found the opposite results (M_low_ = −1.742μV,S.E_.low_ = 0.171;M_high_ = −1.236μV,S.E_.high_ = 0.217) for prevention-focused consumers [$\text{F}\,\left(1,18\right)=8.051,p<0.05,\eta_{\text{P}}^{2}=0.309$]. However, there were no interaction effects of regulatory focus × electrode [$\text{F}\,\left(4,72\right)=0.4,p>0.05,\eta_{\text{P}}^{2}=0.022$], IL × electrode [$\text{F}\,\left(4,72\right)=0.115,p>0.05,\eta_{\text{P}}^{2}=0.006$], or regulatory focus × IL × electrode [$\text{F}\,\left(4,72\right)=0.612,p>0.05,\eta_{\text{P}}^{2}=0.033$].

The mixed repeated measure ANOVA results for the P3 revealed a significant main effect of IL [*F* (1, 18) = 25.765, *p* < 0.01, *η*^2^P = 0.589]: the P3 amplitudes for the low IL condition (*M* = 2.782 μV, S.E. = 0.214) were larger than for the high IL condition (*M* = 1.691 μV, S.E. = 0.123). There were, however, no significant main effect of regulatory focus [*F* (1, 18) = 2.781, *p* > 0.05, *η*^2^P = 0.134] or electrode [*F* (4, 72) = 3.182, *p* > 0.05, *η*^2^P = 0.195]. Importantly, the interaction between IL and regulatory focus was significant [*F* (1, 18) = 27.380, *p* < 0.001, *η*^2^P = 0.965], as shown in Figure 3. A simple effect analysis suggested that the P3 amplitudes were larger for the low IL condition (*M* = 3.616 μV, S.E. = 0.345) than for the high IL condition (*M* = 1.258 μV, S.E. = 0.238) for promotion-focused consumers [*F* (1, 18) = 32.104, *p* < 0.001, *η*^2^P = 0.641]. Notably, there was no difference between the low IL condition (*M* = 1.949 μV, S.E. = 0.184) and high IL condition (*M* = 2.124 μV, S.E. = 0.170) for prevention-focused consumers [*F* (1, 18) = 0.955, *p* > 0.05, *η*^2^P = 0.045]. In addition, the interaction between IL × electrode was significant [*F* (1, 18) = 4.354, *p* < 0.05, *η*^2^P = 0.195]. *Post hoc* comparisons showed that the P3 was more positive in the low IL condition than in the high IL condition over all these electrodes. As expected, there were no interaction effects of regulatory focus × electrode [*F* (4, 72) = 0.378, *p* > 0.05, *η*^2^P = 0.021] or regulatory focus × IL × electrode [*F* (4, 72) = 1.513, *p* > 0.05, *η*^2^P = 0.078].

## Discussion

The present study investigated consumers’ response to online IL from the perspective of regulatory focus by using ERPs. The results showed that the low IL condition led to the reduced RTs, the larger P2 and P3, and an attenuated N2 than the high IL condition for promotion-focused participants, whereas either weakened or even opposite results were observed for prevention-focus participants.

Prior research has shown that the RTs may relate to difficulties in decision-making and cognitive load (Cowen et al., 2002; Wang et al., 2016). Also, the RTs for the matched tasks would be shorter than those for the unmatched jobs (Wang and Wang, 2021). In the current study, promotion-focused consumers may fit more with the low IL condition than the high IL condition. Therefore, shorter RTs were observed in the low IL condition than in the high IL condition for promotion-focused participants. However, the difference between the low and high IL conditions for prevention-focused individuals was not significant. Prior research has suggested that prevention-focused individuals may use accurate decision strategies (Werth and Förster, 2007). In an experiment by Förster et al. (2003), participants were asked to connect the dots in the pictures as quickly and as completely as possible. The results showed that prevention-focused participants may have greater accuracy than promotion-focused participants, suggesting adopting cautious approaches. Following this logic, we supposed that prevention-focused participants would tend to use systematic strategies to improve decision accuracy in both low and high IL conditions. Here, it may result in the undifferentiated RTs toward the low and high IL conditions.

The results of ERP may reflect the neurological process of evaluation of products and purchase decisions. The first stage entailed the early attention mobilization process, as demonstrated by the P2. The P2 is an attention-related component, indicating early automatic allocation of attentional resources (Huang and Luo, 2006; Jing et al., 2019). It has previously been shown that the pursuit of goals in a way that “fits” the individual’s regulatory focus would increase consumers’ attentional resources devoted (Higgins, 2000; Lee and Aaker, 2004; Cesario and Higgins, 2008). In the current study, a decision-making environment with more detailed information may provide a greater regulatory fit with prevention-focused individuals than promotion-focused individuals (Wan et al., 2009). However, the opposite result emerged for those with a promotion focus. Therefore, more attentional resources would be automatically allocated in the low IL condition than in the high IL condition for those with a prevention focus, as reflected by a larger P2 amplitude, whereas the reverse held for those with a promotion focus.

The second stage involved the conflicting information process, as reflected by the N2. In the field of risky decision making, the N2 is sensitive to risky information (Wang et al., 2016). The N2 is also positively correlated with response conflict, depending on the characteristics of the external stimulus and one’s internal state of control (Jin et al., 2017; Shang et al., 2017; Jing et al., 2019). In the present study, those IL conditions that mismatch with their regulatory focus might represent higher risk, which would lead to higher response conflicts and an enhanced N2 amplitude. However, it should be noted that such a conflict effect was distinct from previous research, which examined the matching tasks based on the S1–S2 paradigm (Ma et al., 2007; Mao and Wang, 2008). In these studies, the information from S1 stimuli would be momentarily encoded into the working memory first. When the information from S2 was represented, the interim memory information from S1 would be retrieved and compared with the information from S2. The disparity between the two stimuli resulted in a short-term memory conflict and evoked the N2. Unlike such studies, we examined the matching effect between consumers’ long-term regulatory focus and product IL that we provided. A greater conflict between IL and their regulatory focus would elicit a more negative N2 amplitude. Thus, this type of conflict effect in our study could result from a long-term memory conflict.

The third stage was related to late decision difficulty and confidence assessment, as illustrated by the P3. The less positive P3 would be observed when participants faced greater difficulties or lacked confidence in the decision process (Johnson and Donchin, 1978; Nieuwenhuis et al., 2005). Previous research has shown that the enhanced decision confidence occurred when the self-regulatory orientation matched with their goal pursuit (Higgins et al., 2003; Pham and Avnet, 2004; Wan et al., 2009; Som and Lee, 2012). In the current study, promotion-focused participants may experience greater regulatory fit with a low IL decision environment, whereas prevention-focused participants might experience a greater regulatory fit with a high IL decision environment. Therefore, consumers with a promotion focus would be more confident, and elicit a greater P3 amplitude when faced with the low IL condition. Furthermore, our results also showed that the P3 was larger in the high IL condition compared to the low IL condition for those with a prevention focus. However, the contrast was not significant (*p* > 0.05). Two reasons may account for this result. Firstly, consumers were asked to make purchase decisions under certain time pressures in our study. Therefore, those with a prevention focus would perceive greater decision difficulty and have less confidence to decide in both conditions. Additionally, although we have observed some trends that were consistent with our assumption, relatively few participants may weaken the statistical power in our study.

Our work contributes to research on IL and regulatory focus in several ways. There have been diverging insights on the net effect of IL on consumer decisions in the IL literature. We elucidate the divergent outcomes in the literature by identifying a consumer-level factor. We show that an increased amount of product information is favored only by a segment of consumers (i.e., prevention-focused), while those with a promotion focus favor a small amount of product information. In addition, we also extend the regulatory focus literature by indicating a regulatory fit between regulatory focus and IL, whereas previous studies have focused on the fit between regulatory focus and the number of options presented or the type of information presented.

Furthermore, to open the brain’s black box, our study used ERPs to mine neural mechanisms of how regulatory focus affected the impact of IL on consumers’ online decisions. Most importantly, we found that the P2, N2, and P3 components may indicate a three-stage pattern involving regulatory fit functions in one’s brain. First, people would automatically and expeditiously search for favorite IL conditions, and the more the conditions fit their expectations, the higher the P2 amplitudes. Then, they would judge whether the current amount of product information matched their regulatory focus, and mismatch conditions would evoke the increased memory conflict and a larger N2 amplitude. Finally, consumers would evaluate the decision difficulty in different contexts, as reflected by the P3.

Based on the above findings and discussion, this study provides essential management insights, particularly for product information provision. E-retailers should consider how consumers with different motivations respond to different IL conditions. According to our study, there is no one pattern of IL that will be suitable for all. More specifically, retailers should provide a small amount of product information for promotion-focused consumers; conversely, retailers should offer more product information for those with a prevention focus.

Finally, it is acknowledged that this study has several limitations. First, we only focused on the number of product information without considering its content. Indeed, the concepts embodied by brands are of great importance to purchase decisions (Torelli et al., 2012). For example, some brands tried to deliver the concepts of globalness, reflecting a sense of western meanings, symbols, and values (Alden et al., 1999), others tended to deliver the concepts of cosmopolitanism, representing universalism and the unity of mankind (Steenkamp, 2019). However, no research has explored how consumers respond to the two different brand positioning strategies from the perspective of neuroscience. Likewise, in the context of anti-globalization, consumers may be becoming more conservative and more confident in their own cultures (Guo et al., 2019). Thus, it could be argued that compared to brands that incorporate foreign cultures, those containing local cultures may increase consumers’ confidence in decision making, which may lead to enhanced P3. Second, we only focused on one non-durable product (fruits) in this study. A wider range of non-durable products could be examined in future research to demonstrate the generalizability of our findings. Finally, the N2 is typically maximal at FCz, but we chose five electrodes (F3, F4, FC1, FC2, and Fz) around that location for the N2 analysis because we used a 32-electrode setting (which excludes FCz). We recommend that future studies use a 64-electrode setting to test the generalizability of our study.

## Conclusion

In sum, this study explored the hidden neural mechanism of how regulatory focus influenced the impact of IL on consumers’ online decisions. The behavioral results demonstrated that shorter RTs were observed in the low IL condition than in the high IL condition for those with a promotion focus. However, no significant difference was found for those with a prevention focus. The ERP results indicated that the low IL condition would trigger the enhanced P2 and P3 and an attenuated N2 compared to the high IL condition for promotion-focus consumers, whereas either weakened or even opposite results were found for prevention-focus consumers. We believed that the P2 illustrated the mobilization of attentional resources, the N2 indicated a long-term memory conflict between IL and regulatory focus, and the P3 could be viewed as a reflection of consumers’ decision difficulties and confidence. Generally, these findings suggested that if the IL matched with consumers’ regulatory focus, this fit experience would be conducive to their purchase decision, which may benefit future marketing studies.

## Data Availability Statement

The raw data supporting the conclusions of this article will be made available by the authors, without undue reservation.

## Ethics Statement

The studies involving human participants were reviewed and approved by the School of Economics & Management, Wuyi University. The patients/participants provided their written informed consent to participate in this study.

## Author Contributions

ZX, HH, and MP conceived and designed the experiments and wrote and edited the manuscript. ZX and MP performed the experiments. ZX analyzed the data. All authors contributed to the article and approved the submitted version.

## Conflict of Interest

The authors declare that the research was conducted in the absence of any commercial or financial relationships that could be construed as a potential conflict of interest.

## Publisher’s Note

All claims expressed in this article are solely those of the authors and do not necessarily represent those of their affiliated organizations, or those of the publisher, the editors and the reviewers. Any product that may be evaluated in this article, or claim that may be made by its manufacturer, is not guaranteed or endorsed by the publisher.
