# Corrigendum: To Each Their Own: The Impact of Regulatory Focus on Consumers' Response to Online Information Load

**DOI:** 10.3389/fnins.2022.922758

**Published:** 2022-06-28

**Authors:** Minjing Peng, Zhicheng Xu, Haiyang Huang

**Affiliations:** School of Economics and Management, Wuyi University, Jiangmen, China

**Keywords:** information load, regulatory focus, event-related potentials, P2, N2, P3

In the original article, there was an error. The phrase “Besides, with the conflicts between these physical properties” is corrected with “Besides the conflicts between these physical properties”. This error does not change the main scientific conclusions of the article. A correction has been made to Introduction, Paragraph 8:

“The N2 is a negative-going component with a frontal-central cortex distribution peaking at around 250–350 ms (Folstein et al., 2008). Prior studies indicated that it was relevant to conflict and mismatch (Van Veen and Carter, 2002). For example, more negative N2 amplitudes emerged when the second stimuli did not match the physical characteristics of the first stimuli concerning color or position based on the S1–S2 paradigm (Wang et al., 2004; Mao and Wang, 2008). Besides the conflicts between these physical properties, the N2 could also be elicited by perception conflicts (Ma et al., 2007). For example, a higher cognitive conflict would be observed in the counter-conformity decisions, and then a larger N2 amplitude would be evoked (Gajewski et al., 2016). Conversely, Shang et al. (2017) suggested no conflict would be produced when consumers perceived a more excellent brand extension fit, which can be revealed in a smaller N2 amplitude. In addition, Achtziger et al. (2014) showed that participants who over-valued new information in the belief-updating economic decisions were less sensitive to conflict detection, as reflected by the N2. According to regulatory fit, consumers would produce a sense of fluency and perceive a smaller decision conflict when the task at hand matches with individuals' regulatory focus (Sellier and Chattopadhyay, 2009). Thus, we assume that more cognitive conflicts will be caused and elicit a larger N2 amplitude in the decision process if IL mismatches with consumers' regulatory focus. More specifically, the high IL condition will induce a larger N2 amplitude compared to the low IL condition for promotion-focused consumers. In contrast, the opposite results will be found for prevention-focused consumers.”

In the original article, there was an error. The phrase “in the IL condition” is corrected with “in the high IL condition”. This error does not change the main scientific conclusions of the article. A correction has been made to Materials and Methods, Materials and Pretest, Paragraph 2:

“Based on previous studies (Sicilia and Ruiz, 2010), we developed two versions of the material to manipulate the IL condition. Specifically, the ultimate IL for each condition (six for the low IL condition and twelve for the high IL condition) was established through a pretest. In this pretest (*n* = 201), we used a 5-point Likert scale adapted from Lee and Lee (2004) to determine the level of perceived IL (i.e., “There were many characteristics of fruits to consider”). An independent-sample *t*-test indicated a significant difference [*M*_*low*_ = 2.90 vs. *M*_*high*_ = 3.85; t(199) = −15.50; *p* < 0.001] in perceiving IL levels between the two IL conditions, which suggested that there is more information needed to be addressed for participants in the high IL condition. All pictures were processed to maintain consistency in text style, lightness, and saturation.”

In the original article, there were some incorrect math symbols. The math symbol “η^2^P 0.243”, “η^2^P < 0.222”, “η^2^P < 0.612”, “*p* < 0.05” and “η^2^P < 0.052” is corrected with “η^2^P = 0.243”, “‘η^2^P = 0.222”, “η^2^P = 0.612”, “*p* > 0.05” and “η^2^P = 0.052” respectively. A correction has been made to Results, Behavioral Data, Paragraph 1:

“A two-way 2 (IL: low vs. high) × 2 (regulatory focus: promotion vs. prevention) mixed repeated measure ANOVA was performed for the response times (RTs). We used SPSS 25.0 for statistical tests. The results demonstrated a significant main effect of IL [*F* (1, 18) = 28.791, *p* < 0.001, η^2^P = 0.113]: the RTs for the high IL condition (*M* = 1,682 ms, *SD* = 46) were longer than the low IL condition (*M* = 1,595 ms, *SD* = 44). Furthermore, the main effect of regulatory focus was significant [*F* (1, 18) = 5.787, *p* < 0.05, η^2^P = 0.243]: the RTs for prevention-focused individuals (*M* = 1,786 ms, *SD* = 66) were longer than for promotion-focused individuals (*M* = 1,491 ms, *SD* = 84). Importantly, the interaction between IL and regulatory focus was also significant [*F* (1, 18) = 5.147, *p* < 0.05, η^2^P = 0.222]. A simple effect analysis showed that the RTs for the low IL condition (*M* = 1,420 ms, *SD* = 78) were significantly shorter than those for the high IL condition (*M* = 1,563 ms, *SD* = 91) for promotion-focused consumers [*F* (1, 18) = 28.332, *p* < 0.001, η^2^P = 0.612], while the contrast between the low IL condition and the high IL condition for prevention-focused consumers was not significant [*F* (1, 18) = 0.979, *p* > 0.05, η^2^P = 0.052].”

In the original article, there was an incorrect math symbol. The math symbol “F (1, 18) 2.781” is corrected with “F (1, 18) = 2.781”. A correction has been made to Results, Event-Related Potential Data, Paragraph 4:

“The mixed repeated measure ANOVA results for the P3 revealed a significant main effect of IL [*F* (1, 18) = 25.765, *p* < 0.01, η^2^P = 0.589]: the P3 amplitudes for the low IL condition (*M* = 2.782μV, S.E. = 0.214) were larger than for the high IL condition (*M* = 1.691 μV, S.E. = 0.123). There were, however, no significant main effect of regulatory focus [*F* (1, 18) = 2.781, *p* > 0.05, η^2^P = 0.134] or electrode [*F* (4, 72) = 3.182, *p* > 0.05, η^2^P = 0.195]. Importantly, the interaction between IL and regulatory focus was significant [*F* (1, 18) = 27.380, *p* < 0.001, η^2^P = 0.965], as shown in **Figure 3**. A simple effect analysis suggested that the P3 amplitudes were larger for the low IL condition (*M* = 3.616 μV,S.E. = 0.345) than for the high IL condition (*M* = 1.258 μV, S.E. = 0.238) for promotion-focused consumers [*F* (1, 18) = 32.104, *p* < 0.001, η^2^P = 0.641]. Notably, there was no difference between the low IL condition (*M* = 1.949 μV, S.E. = 0.184) and high IL condition (*M* = 2.124 μV, S.E. = 0.170) for prevention-focused consumers [*F* (1, 18) = 0.955, *p* > 0.05, η^2^P = 0.045]. In addition, the interaction between IL × electrode was significant [*F* (1, 18) = 4.354, *p* < 0.05, η^2^P = 0.195]. *Post hoc* comparisons showed that the P3 was more positive in the low IL condition than in the high IL condition over all these electrodes. As expected, there were no interaction effects of regulatory focus × electrode [*F* (4, 72) = 0.378, *p* > 0.05, η^2^P = 0.021] or regulatory focus × IL × electrode [*F* (4, 72) = 1.513, *p* > 0.05, η^2^P = 0.078].”

The authors apologize for those errors and state that those do not change the scientific conclusions of the article in any way. The original article has been updated.

## Publisher's Note

All claims expressed in this article are solely those of the authors and do not necessarily represent those of their affiliated organizations, or those of the publisher, the editors and the reviewers. Any product that may be evaluated in this article, or claim that may be made by its manufacturer, is not guaranteed or endorsed by the publisher.
